# Decreased costs and retained QoL due to the ‘PACE Steps to Success’ intervention in LTCFs: cost-effectiveness analysis of a randomized controlled trial

**DOI:** 10.1186/s12916-020-01720-9

**Published:** 2020-09-22

**Authors:** Anne B. Wichmann, Eddy M. M. Adang, Kris C. P. Vissers, Katarzyna Szczerbińska, Marika Kylänen, Sheila Payne, Giovanni Gambassi, Bregje D. Onwuteaka-Philipsen, Tinne Smets, Lieve Van den Block, Luc Deliens, Myrra J. F. J. Vernooij-Dassen, Yvonne Engels, on behalf of the PACE trial group, Paula Andreasen, Ilona Barańska, Catherine Bassal, Danni Collingridge Moore, Harriet Finne-Soveri, Katherine Froggatt, Teija Hammar, Rauha Heikkilä, Jo Hockley, Elisabeth Honinx, Hein van Hout, Violetta Kijowska, Maud Ten Koppel, Outi Kuitunen-Kaija, Suvi Leppäaho, Federica Mammarella, Martina Mercuri, Rose Miranda, Emilie Morgan de Paula, Nele Van Den Noortgate, Mariska Oosterveld-Vlug, Agnieszka Pac, H. Roeline W. Pasman, Sophie Pautex, Sheila Payne, Ruth Piers, Lara Pivodic, Paola Rossi, Katarzyna Szczerbińska, Ivan Segat, Jenny T. van der Steen, Agata Stodolska, Marc Tanghe

**Affiliations:** 1 IQ Health Care, Radboud Institute for Health Sciences, Radboud University Medical Centre, Nijmegen, The Netherlands; 2 Department of Anaesthesiology, Pain and Palliative Medicine, Radboud University Medical Centre, P.O. Box 9101, 6500 HB Nijmegen, The Netherlands; 3 Department for Health Evidencef, Radboud University Medical Centre, Nijmegen, The Netherlands; 4 Unit for Research on Aging Society, Epidemiology and Preventive Medicine Chair, Jagiellonian University Medical College, Kraków, Poland; 5 National Institute for Health and Welfare, Helsinki, Finland; 6 Division of Health Research, Lancaster University, Lancaster, England; 7 Faculty of Medicine and Surgery, Fondazione Policlinico Universitario A. Gemelli, IRCCS and Università Cattolica del Sacro Cuore, Rome, Italy; 8 Department of Public and Occupational Health, Amsterdam Public Health Research Institute, Amsterdam UMC, Vrije Universiteit Amsterdam, Amsterdam, The Netherlands; 9 End-of-Life Care Research Group, Vrije Universiteit Brussel & Ghent University, Brussel, Belgium

**Keywords:** Cost-benefit analysis, Palliative care, Nursing homes, Teaching

## Abstract

**Background:**

The number of residents in long-term care facilities (LTCFs) in need of palliative care is growing in the Western world. Therefore, it is foreseen that significantly higher percentages of budgets will be spent on palliative care. However, cost-effectiveness analyses of palliative care interventions in these settings are lacking. Therefore, the objective of this paper was to assess the cost-effectiveness of the ‘PACE Steps to Success’ intervention. PACE (Palliative Care for Older People) is a 1-year palliative care programme aiming at integrating general palliative care into day-to-day routines in LTCFs, throughout seven EU countries.

**Methods:**

A cluster RCT was conducted. LTCFs were randomly assigned to intervention or usual care. LTCFs reported deaths of residents, about whom questionnaires were filled in retrospectively about resource use and quality of the last month of life. A health care perspective was adopted. Direct medical costs, QALYs based on the EQ-5D-5L and costs per quality increase measured with the QOD-LTC were outcome measures.

**Results:**

Although outcomes on the EQ-5D-5L remained the same, a significant increase on the QOD-LTC (3.19 points, *p* value 0.00) and significant cost-savings were achieved in the intervention group (€983.28, *p* value 0.020). The cost reduction mainly resulted from decreased hospitalization-related costs (€919.51, *p* value 0.018).

**Conclusions:**

Costs decreased and QoL was retained due to the PACE Steps to Success intervention. Significant cost savings and improvement in quality of end of life (care) as measured with the QOD-LTC were achieved. A clinically relevant difference of almost 3 nights shorter hospitalizations in favour of the intervention group was found. This indicates that timely palliative care in the LTCF setting can prevent lengthy hospitalizations while retaining QoL. In line with earlier findings, we conclude that integrating general palliative care into daily routine in LTCFs can be cost-effective.

**Trial registration:**

ISRCTN14741671.

## Background

Health care costs rise while resources remain limited. Therefore, providing evidence on health economic evaluations to guide decisions is increasingly important, especially in palliative care and in long-term care facilities (LTCFs) [1], because the need for long-term institutional care for older people is growing as a result of the great leap in life expectancy achieved in the twentieth century in Western societies and the related increase in prolonged and more complex illness trajectories [2–4]. Consequently, a significant number of frail older people live in LTCFs and will develop palliative care needs as they approach the end of life. At the same time, a number of descriptive studies show that palliative care for this patient group can be suboptimal: it is not always available, symptoms appear under-estimated and burdensome treatments are continued or initiated without having explored residents’ individual preferences [5–9]. Furthermore, potentially avoidable hospital admissions are common among LTCF residents [10].

According to the Economist Intelligence Unit, the growing number of people that might benefit from palliative care will significantly increase the percentage of budgets spent on palliative care [11]. There are indications that palliative care interventions are less costly than usual care or even cost-effective [12]. However, mostly home, hospital and hospice-based palliative care programs have been evaluated [12–16]. Therefore, evidence on the cost-effectiveness of palliative care interventions specifically in LTCFs is needed. In our study, the EU-funded PACE (Palliative Care for Older People) Steps to Success intervention, aimed at training LTCF staff to deliver palliative care to residents, was provided [17]. Van den Block et al. showed that residents’ comfort in the last week of life did not change, and that staff had statistically significantly better knowledge of palliative care [18].

In this study, new evidence regarding effects on costs and quality of end of life (care) due to the PACE Steps to Success intervention is introduced. It aimed to evaluate the cost-effectiveness of the intervention throughout seven counties.

## Methods

### Study design

This study was part of the EU PACE project [19]. In PACE, a cluster randomized controlled trial (cRCT) of a palliative care intervention for residents in LTCFs was conducted in Belgium (Flanders), Finland, Italy, Netherlands, Poland, England and Switzerland. In each country, LTCFs were randomly assigned to either the intervention condition or to usual care, using stratified randomization. Pre-specified outcome measures in the cost-effectiveness analysis were direct medical costs, quality-adjusted life years (QALYs) based on the EuroQol-5D-5L (EQ-5D-5L) and costs per quality increase as measured with a specific end of life questionnaire (QOD-LTC). As suggested by the EQUATOR Network, the CHEERS guideline for economic evaluations was used [20].

The PACE Steps to Success intervention was implemented over a 12-month period between 2016 and 2017. Respondents gave informed consent in writing prior to filling in questionnaires. In Poland and the Netherlands, this was not required, as questionnaires were filled in anonymously. The study protocol was approved by the relevant ethics committees in all participating countries and was registered at www.isrctn.com – ISRCTN14741671 (FP7-HEALTH-2013-INNOVATION-1 603111) on July 30, 2015. This study was supported by the EU 7th Framework Program, grant agreement 6031111. The funding organization was not involved in the study design, collection, analysis and interpretation of the data nor in the decision to approve publication of this manuscript.

### Participants

In each country, researchers randomly selected LTCFs (clusters) from predefined regions. For eligibility criteria, see the PACE protocol paper [19]. In each LTCF, residents who had died over the previous 4-month period were listed.

### Intervention

The ‘PACE Steps to Success’ intervention aims at integrating general palliative care into day-to-day routines in LTCFs by means of a train-the-trainer approach [19]. The intervention is based on the ‘Route to Success in Long-term Care Facilities’, developed in the UK [17]. Table 1 shows the six steps of the PACE Steps to Success intervention. It was hypothesized that through the training of facility staff, residents would receive high-quality palliative care, which in turn would improve the quality of end of life of these residents. LTCFs assigned to usual care were allowed to use all (supportive) services without restriction.

**Table 1:** The six steps of the ‘PACE Steps to Success intervention’

Step	Content of the step
**Discussions as the end-of-life approaches**	Advance care planning discussions with residents and/or families are conducted to elicit wishes and preferences around end-of-life care.
**Assessment, care planning and review**	A ‘Mapping Changes in Condition chart’ is used monthly by nurses and care assistants to plot changes (deterioration and improvement) in a resident’s physical condition.
**Coordination of care**	Using a Palliative Care Register, residents who are identified as expected to live less than 6 months are discussed in detail during monthly multidisciplinary review meetings. A summary sheet is sent to physicians who were not able to attend the meeting.
**Delivery of high-quality care**	Staff learns about symptom control and complex communication skills, with a focus on pain and depression.
**Care in the last days of life**	The Last Days of Life checklist prompts and guides the care in the last days of life, with a focus on recognizing dying, communication with family, regular assessment of symptoms, anticipatory medication prescription, hydration, and psychosocial and spiritual support.
**Care after death**	Reflective meetings following a death are held to support staff and encourage experiential learning.

### Data collection and instruments

The cost-effectiveness of the PACE ‘Steps to Success’ intervention was assessed from a health care perspective, including direct medical and intervention-related costs. A cross-sectional study of resident deaths in participating LTCFs was conducted. LTCFs reported deaths of residents during the previous 4 months, irrespective of the place of death. For each case, multiple structured after-death questionnaires were filled in by a staff member most involved (preferably a nurse, otherwise a care assistant) and a relative at baseline (T0), and post-intervention at month 13 (T1) and month 17 (T2). See flowchart in Additional file 1: Fig. S1.

Since the impact of the intervention on both costs and effects was anticipated to mainly take place in the terminal phase of life, a time horizon of 1 month was chosen. Questions regarding care services received, which could be validated by administrative data (filled in by a staff member) and quality of life (QoL) during the last month of life (filled in by a staff member and a relative), were used to determine effects and direct medical resource use on a per resident basis. Using data from proxy respondents is a commonly employed approach in studies of this type and appears to be an acceptable source of data [21, 22].

#### Costs

Quantities of resources used in the last month of life (hospital admissions, visits of health care professionals, received intensive treatments like CPR or surgery (yes or no), see Additional file 2: Table S1) were multiplied by standard unit costs in euros. These unit costs were based on reference prices from 2017, the year the intervention was provided, calculated by the Dutch National Health Care Institute (ZN) and average per unit sales prices established by the Dutch Health Care Authority (NZA). If prices were only available for other years, they were adjusted using the consumer price index published by Statistics Netherlands [23]. If they were not available, health insurer contract rates were used. By using standard unit costs, the price parameter was kept constant over countries, so that results of the cost-effectiveness analysis could be explained by variances in volumes resource use only. See Additional file 2: Table S1 for the clustered care units that were used to measure resource use, including tariffs.

Mean costs associated with the intervention (hours dedicated to the intervention of coordinators and trainers, materials, accommodation) were identified and calculated back to resident level by multiplying the LTCFs’ number of beds by the occupancy rate. See Additional file 2: Fig. S2. In this calculation, the turnover rate of residents was considered. Since little is known about the sustainability of complex interventions in long-term care [24], a comprehensive discussion within the PACE consortium was held. As a conclusion, a depreciation period of 3 years was chosen as base-case. Subsequently, sensitivity analyses to determine the impact of the depreciation period were conducted in which it was assumed the effect of the intervention would wear off a shorter period of time.

#### Effects

The 5-level EQ-5D version (EQ-5D-5L) and the Quality of Dying in Long-Term Care (QOD-LTC) were used to measure intervention effects. The EQ-5D-5L measures five health-related QoL domains: mobility, self-care, usual activities, pain/discomfort and anxiety/depression. Preferences for the EQ-5D-5L were based on value sets from England [25, 26] and were elicited by means of both time trade-off and discrete choice experiments [26]. The use of the EQ-5D is recommended by international guidelines on economic evaluation [27, 28], making it the most often used outcome measure in economic evaluations in health care, increasing the comparability of results across literature. As suggested in earlier work [29], in this study, a specific QoL instrument for palliative care was used in addition to the EQ-5D. The QOD-LTC covers both quality of care (64%) and quality of dying (36%) items, measures psychosocial quality of end of life (care) and comprises three subscales: personhood, closure and preparatory tasks [30].

### Statistical analyses

Differences in both costs and effects were analysed by means of regression analyses, with LTCF as the cluster. A mixed model approach was used, as this is able to capture the design complexity and correlation structures. Cost-effectiveness was integrated in one outcome measure, the net monetary benefit (NMB), and used as a dependent variable in the mixed model [31]. The NMB is a summary statistic representing the value of the intervention in monetary terms when a willingness-to-pay for a unit of effect, such as the QALY, is assumed. If the net value of the benefits outweighs the costs, the investment is said to increase health. If a reduction in costs at equal effects is realized, cost-minimization is pursued.

Covariates considered were age, gender, disease severity (measured with the Bedford Alzheimer Nursing Severity Scale – BANS-S, range 7–28), baseline measurement and the treatment dummy (intervention vs. control). Baseline measurement was taken into account to control for differences on baseline in case mix (regarding characteristics of residents) between LTCFs, both between and within countries. Since costs normally are right skewed—there are no negative costs, and some patients incur high costs (outliers)—a non-parametric bootstrap, in which we randomly sampled with replacement, was performed. This approach avoids the need to make assumptions about the shape of the distribution but instead uses the observed distributions of the studies’ cost data [32]. Analyses were on an intention-to-treat and complete case analysis was pursued.

## Results

Deceased residents from 73 LTCFs^1^ from seven countries were identified. Concerning deceased residents, we collected 551 of 610 questionnaires from staff at baseline (90.3%) and 984 of 1178 post-intervention (83.5%) in 37 intervention and 36 control homes. Two hundred fifty-nine of 467 questionnaires from relatives were collected at baseline (55.5%) and 498 of 939 post-intervention (53.0%). We report on findings from both groups but focus on findings from staff questionnaires because of the considerably higher response rate. In addition, within the European context, nurses’ perceptions seem to align more closely with those of patients than perceptions of relatives do [33, 34].

The mean age of the deceased residents was 85.7 years, with a BANS-S disease severity score of 19.1 (range 7–28), and 65% was female. Resident characteristics in intervention and control LTCFs at baseline (T0) and post-intervention (T1 + T2) were broadly comparable. See Table 2.

**Table 2:** Resident characteristics control and intervention LTCFs on baseline and post-intervention

	Baseline (T0)		Post-intervention (T1 + T2)	
	Control (*n* = 272)	Intervention (*n* = 279)	Control (*n* = 558)	Intervention (*n* = 425)
Mean age	85.22	85.68	85.58	85.91
% female	70.6	60.6	64.7	64.0
BANS-S	19.93	19.03	18.95	18.75
% dementia	71.8	70.3	71.8	66.8

### Costs

Between baseline and post-intervention, costs in the last month of life increased by €600.75 for residents dying in control LTCFs and decreased by €257.52 for residents dying in LTCFs receiving the PACE Steps to Success intervention. See Table 3. After bootstrapping and controlling for age, gender, residents’ disease severity, country, baseline measurement and group, a significantly lower cost of €983.23 for residents dying in intervention LTCFs was found. See Table 4. This decrease in cost was mainly due to significantly lower costs related to hospital admission (− €919.51, see Additional file 3: Table S2). This decrease can be explained by a combination of shorter length of hospitalizations and the type of wards residents were admitted to, although both elements did not decrease significantly on their own. Of those residents admitted, between pre- and post-test admission decreased with 2.2 nights in the intervention group and increased with 0.7 nights in the control group (a difference of 2.9 nights in favour of the intervention group). Moreover, mainly costs related to hospitalizations on the geriatric, general and ICU ward decreased (80.6% of total cost decrease). The above figures are based on a 3-year depreciation period, resulting in intervention-related costs of €124.08 per resident in the intervention arm. When bringing back the depreciation period to 1 year, the intervention still brought about significantly lower costs (− €934,84, 95% CI − €1713,78 to − €273,02, *p* value 0.023).

**Table 3:** Mean costs resource use and quality of end of life effects last month of life for control and intervention LTCFs on baseline and post intervention (unadjusted)

	Baseline (T0)		Post-intervention (T1 + T2)	
	Control (*n* = 272)	Intervention (*n* = 279)	Control (*n* = 558)	Intervention (*n* = 425)
Mean costs resource use	€1,361.89	€1,667.87	€1,962.64	€1,410.35
EQ-5D-5L (range 0–1)	0.159^a^	0.210^a^	0.196^a^	0.186^a^
	0.155^b^	0.130^b^	0.160^b^	0.160^b^
QOD-LTC (range 11–55)	38.24	37.84	38.34	40.96

^a^Staff member (nurse or care assistant) most involved

^b^Relative

**Table 4:** Adjusted mean differences in costs resource use and quality of end of life effects last month of life, incl. 95% CIs and *P* values

	Baseline (T0)			Post-intervention (T1 + T2)		
	Mean difference	95% CI	*P* value	Mean difference	95% CI	*P* value
Mean costs resource use	€382.56	− €240.29 to €1136.67	0.15	− €983.28	− €1,762.22 to − €321.46	**0.02**
EQ-5D-5L (range 0–1)	0.035	− 0.186–0.884	0.20	− 0.038	0.087–0.011	0.13
QOD-LTC (range 11–55)	− 1.09	− 3.15–0.96	0.29	3.19	1.72–4.65	**0.00**

### Effects

After bootstrapping and controlling for age, gender, residents’ disease severity, country, baseline measurement and group, no significant differences in quality of end of life as measured with the EQ-5D-5L were found between intervention and control LTCFs between baseline and post-intervention. The QOD-LTC significantly improved for residents dying in LTCFs receiving the intervention (3.19, 95% CI 1.72–4.65, *p* value 0.00). See Tables 3 and 4. The fact both measures show a low correlation (Pearson’s coefficient, 0.14) confirms the relevance of not only measuring HR-QoL through the EQ-5D-5L when doing CEAs in palliative care, as suggested in earlier work [29].

### Cost-effectiveness

The PACE Steps to Success intervention dominated the common practice alternative (control). It appeared both cheaper (− €983,28, *p* 0.02) and more effective on the QOD-LTC or not significantly different on the EQ-5D-5L. See Table 4. The NMB over the total relevant willingness-to-pay range for a QALY gained, from €20.000 to €80.000, in this study is therefore always bigger than zero, meaning added value. Because no significant differences were found on the EQ-5D-5L, the NMB was not considered in a mixed model as the decision rule changed to cost-minimization.

## Discussion

Costs decreased and QoL was retained due to the PACE Steps to Success intervention. The 1-year palliative care programme realized significant cost savings and improved quality of end of life (care) in the last month of life (QOD-LTC), meaning it strengthened sense of personhood, closure and preparatory tasks. Outcomes on the EQ-5D-5L were unchanged. Cost savings mainly resulted from decreased hospitalization-related costs. A clinically relevant difference of almost 3 nights shorter (from 9 to 6 nights; a decrease of 30%) hospitalizations in favour of the intervention group was found, indicating residents are returning to their LTCF earlier. Mainly costs related to hospitalizations on the geriatric, general and ICU ward decreased (80.6% of total cost decrease). Although the length of hospitalization and the cost decrease on the mentioned wards did not decrease significantly, the *combination* of both components of the composite outcome pointing in the same direction resulted in a significant cost decrease. 

The decrease on these specific wards plausibly is not by chance. Patients entering the hospital normally are screened on the general ward, and on the geriatric ward, the desirability of care is discussed. In our study, this work was already done in the LTCF through the PACE intervention. Regarding the decrease on the ICU, the discussions with residents and families to elicit wishes and preferences around EoL as part of the PACE intervention in the LTCF presumably made residents and staff not prefer intensive hospital care anymore, mostly as the condition of the resident is too frail to profit from such intensive care and might even be harmful. A recent review showed that comparable interventions are found to decrease life-sustaining treatment and prevent hospitalization [35]. A JAMDA review found that in the nursing home population, ACP decreased hospitalization rates up to a quarter [36]. In line with these findings, we therefore conclude that investing in palliative care training for LTCF staff can increase quality of care and the last phase of life and reduce direct medical costs.

Concerning the effects on the cost side, our finding that the PACE Steps to Success intervention influences costs related to hospitalizations in the last month of life is in line with earlier research showing hospital admission in the last phase of life generally speaking are found to be undesirable, as patients mostly prefer less intensive treatment and desire comfort care during the end of life [37, 38]. One study even showed that higher health care costs in the last phase of life were associated with worse quality of death [39]. Also from a health care perspective, this finding is highly relevant as potentially avoidable hospital admissions are common among LTCF residents [10], and costs involved with hospital admissions typically represent a considerable percentage of total spending in the last months of life [40]. Nevertheless, there seems to be room for improvement as the number of hospitalizations and intensive treatments did not decrease. Further research is needed focusing on identifying those residents most at risk for hospitalizations and decreasing those that are avoidable.

Moreover, with regard to the impact on the effect side, the realized improvement on the QOD-LTC, measuring both items related to quality of care and quality of dying [41], evidently is highly desirable as palliative care for the subject patient group can be suboptimal, and opportunities for improvement in terms of QoL in the last phase of life in LTCFs are notable [5–9]. As communication, assessment and care planning are core components of the PACE Steps to Success intervention, the significant improvement on the QOD-LTC is plausible. Since no significant differences were found on the EQ-5D-5L, utilities are not available for the QOD-LTC, and survival effects were not to be expected [42], QALYs were not calculated in this study. If survival would have been influenced though, this was missed.

### Strengths and weaknesses

This pioneering large-scale study is the first to internationally consider the cost-effectiveness of a palliative care intervention in LTCFs. As suggested in earlier work [29], a specific QoL instrument for palliative care in the form of the QOD-LTC was used in addition to the EQ-5D-5L as the latter is mainly concerned with health and the recovery of health and contains domains often seen as less relevant in the context of palliative care, in which values such as patient dignity, spiritual and psychosocial wellbeing and bereavement support are central. Another strength is the homogeneous study group: only data concerning the last weeks of life of residents were used.

In earlier research, it was argued that using backward counting to chart health care utilization might not give full insight in costs involved [43], as patients receiving palliative care might live longer, resulting in higher costs for additional care which might eclipse the savings accrued through lower intensity of care at the end of life [44–46]. However, these findings could not be replicated in recent systematic reviews [42, 47]. Moreover, the fact our study was assessed from a health care perspective, excluding informal care costs such as out-of-pocket payments of relatives and costs associated with loss in productivity (such as absenteeism), plausibly has not caused considerable bias as presumably fairly limited informal care costs (e.g. out-of-pocket expenses and productivity losses) are involved with residents living in LTCFs. As the focus was on *volume* changes, not on *price* effects, the fixed tariffs for care were used. Nevertheless, when using a general tariff for nights spent in the hospital, a significant cost decrease was still found. However, national profits evidently differed per country, as for example labour cost is lower in Poland than the Netherlands. Combining of post-intervention data (at T1 and T2) because of a lower-than-expected response rate did not influence our findings: significant cost-savings were found on both time points.

The fact treatments received had to be dichotomized is a limitation though, as it was not captured if residents had multiple same treatments. Moreover, value sets from England were used to achieve utility scores for the EQ-5D-5L—ranging from 0 (death) to 1 (perfect health)—since value sets were not available for all participating countries [25, 26]. It would be valuable for future studies to be able to use population-specific valuation sets better reflecting cultural differences and so increase validity of the measurement [48]. Also, the UK National Institute for health and Care Excellence (NICE) does not recommend using the EQ-5D-5L value set [49], because of concerns regarding its quality and reliability [50, 51]. Furthermore, using a 3-month period has been tested in previous research and has been shown to limit recall bias [7, 52–54]. In the PACE study, this period was lengthened to 4 months when LTCFs exceptionally did not meet minimal numbers of inclusion. Because of this, some memory bias might have occurred. Additionally, data were collected retrospectively over the last month of life in the PACE project [2]. Therefore, sensitivity analysis considering longer time horizons was not possible. Lastly, respondents were not blinded post-intervention. This might have influenced their responses, as staff may have wanted to report better quality after the intervention. However, the fact the study did not find improvements on staff-reported comfort in the last week of life in the intervention group [18] suggests that this will have been limited.

### Generalizability and future research

Although a pattern of palliative care interventions being less costly than their comparators was also recently found in a review study [12], relatively little is known on the cost-effectiveness of palliative care, especially within LTCFs. Only O’Sullivan et al. on a national level looked at this specific setting and in line with our findings saw a significant decrease in hospitalization related costs [55]. Future research could look into possibilities of using better fitting QoL measurement tools—for example building on the capabilities approach [56]—for patient groups for which health-related QoL outcomes are too narrow [29, 57], and calculating utilities for these tools. Moreover, we suggest looking into possibilities of investing savings due to interventions like ‘PACE Steps to Success’ in quality palliative care in LTCFs.

## Conclusions

Costs decreased and QoL was retained due to the PACE Steps to Success intervention. This study showed that the PACE Steps to Success intervention is cost-effective. Direct medical costs significantly decreased, mainly due to significantly lower hospitalization related costs (− €983.28, 95% CI − €1762.22 to − €321.46, *p* value 0.02) and quality of end of life (care) in the last month of life (QOD-LTC) significantly improved (3.19, 95% CI 1.72–4.65, *p* value 0.00). There is room for improvement though. Further zooming in on which hospitalizations are justifiable and which are not is recommended. Furthermore, rolling out the intervention by continuous training might also positively impact the number of hospitalizations and intensive treatments in the last month of life.

## Supplementary information

**Additional file 1 Fig. S1:** Flowchart PACE cluster RCT.

**Additional file 2 Table S1.** Care units tariffs used. **Fig. S2.** Calculation mean intervention costs.

**Additional file 3 Table S2.** Cost differences per care cluster.

## Data Availability

Deidentified participant data are available for research purposes on request by the PACE consortium.

## Abbreviations

BANS-S: Bedford Alzheimer Nursing Severity Scale
EQ-5D-5L: EuroQol-5D-5L
LTCFs: Long-term care facilities
NMB: Net monetary benefit
NZA: Dutch Health Care Authority
PACE: Palliative Care for Older People (PACE)
QALY: Quality-adjusted life year
QOD-LTC: Quality of Dying in Long-Term Care (QOD-LTC)
QoL: Quality of life (QoL)
ZN: Dutch National Health Care Institute

## Footnotes

1 Of the 78 recruited LTCFs, three dropped out; one had no deaths to report on, and one returned no questionnaires post-intervention.
