# Supplementary figures and images for: Decreased costs and retained QoL due to the ‘PACE Steps to Success’ intervention in LTCFs: cost-effectiveness analysis of a randomized controlled trial
Source: BMC Med. 2020 Sep 22;18:258. doi: 10.1186/s12916-020-01720-9 (PMC7507669; doi:10.1186/s12916-020-01720-9)

**ADDITIONAL FILE 1**

**Figure S1. Flowchart PACE cluster RCT**

*
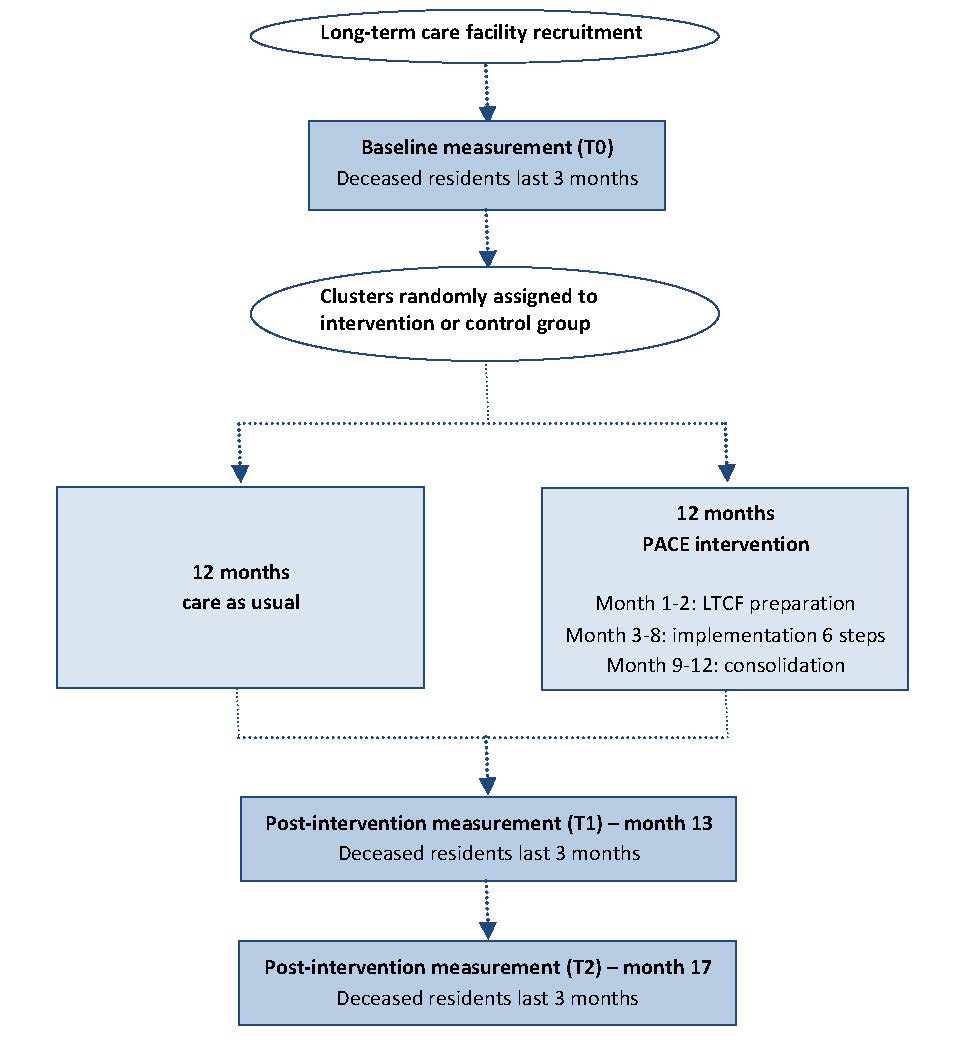
*

Supplement: Supplementary file 1 — Additional file 1 Fig. S1: Flowchart PACE cluster RCT. [file 12916_2020_1720_MOESM1_ESM.docx]
