# Supplementary material for: Decreased costs and retained QoL due to the ‘PACE Steps to Success’ intervention in LTCFs: cost-effectiveness analysis of a randomized controlled trial
Source: BMC Med. 2020 Sep 22;18:258. doi: 10.1186/s12916-020-01720-9 (PMC7507669; doi:10.1186/s12916-020-01720-9)
Supplement: Supplementary file 2 — Additional file 2 Table S1. Care units tariffs used. Fig. S2. Calculation mean intervention costs. [file 12916_2020_1720_MOESM2_ESM.docx]

**ADDITIONAL FILE 2**

| Care unit | | Tariff | Source |
| --- | --- | --- | --- |
| Hospital admission | Night geriatric ward | €803,33 | NZA |
|  | Night psychiatric ward | €302,00 | ZN |
|  | Night internal medicine | €359,00 | NZA |
|  | Night surgery ward | €405,00 | ZN |
|  | Night neurology ward | €395,00 | ZN |
|  | Night general ward | €476,00 | ZN |
|  | Night intensive care unit | €1.186,00 | ZN |
|  | Emergency unit | €259,00 | ZN |
|  | Palliative care unit | €3.900,00 | NZA |
| Visit health care professional | General practitioner | €50,00 | ZN |
|  | Geriatrician | €91,00 | ZN |
|  | Neurologist | €99,00 | ZN |
|  | Psychiatrist | €94,00 | ZN |
|  | Occupational therapist | €33,00 | ZN |
|  | Social worker | €65,00 | ZN |
|  | Psychologist | €64,00 | ZN |
|  | Physiotherapist | €33,00 | ZN |
| Treatments (yes or no) | CPR | €539,47 | Insurer |
|  | Artificial ventilation | €462,59 | Insurer |
|  | Blood transfusion | €853,68 | Insurer |
|  | Chemo or radiotherapy | €1.786,07 | NZA |
|  | Surgery | €6.230,00 | NZA |

**Table S1. Care unit tariffs used**

Figure S2. Calculation mean intervention costs
