# Supplementary material for: Decreased costs and retained QoL due to the ‘PACE Steps to Success’ intervention in LTCFs: cost-effectiveness analysis of a randomized controlled trial
Source: BMC Med. 2020 Sep 22;18:258. doi: 10.1186/s12916-020-01720-9 (PMC7507669; doi:10.1186/s12916-020-01720-9)
Supplement: Supplementary file 3 — Additional file 3 Table S2. Cost differences per care cluster. [file 12916_2020_1720_MOESM3_ESM.docx]

**ADDITIONAL FILE III**

**Table S2. Cost differences per care cluster**

| *Time group *  facility condition* | Cost difference | Sig. (2-tailed) | 95%-CI | |
| --- | --- | --- | --- | --- |
|  |  |  | *Lower* | *Upper* |
| Hospital admissions | ­ €919,51 | **0.018** | ­ €1.725,97 | ­ €299,56 |
| Intensive treatments | - €138,95 | 0.202 | - €333,72 | €19,43 |
| Visits health care  professionals | €92,93 | 0.268 | - €70,37 | €272,61 |
